# Psychometric evaluation of the Farsi version of the diabetes foot self-care bahavior scale

**DOI:** 10.1186/s13047-020-00437-5

**Published:** 2020-11-30

**Authors:** Ali Hasanpour Dehkordi, Yen-Fan Chin, Tzu-Ting Huang, Abbas Ebadi, Reza Ghanei Gheshlagh

**Affiliations:** 1 Social Determinants of Health Research Center, School of Allied Medical Sciences, Shahrekord University of Medical Sciences, Shahrekord, Iran; 2 School of Nursing, College of Medicine, Chang Gung University, Taoyuan City, Taiwan; 3 Healthy Aging Research Center, and School of Nursing, Chang Gung University, Taoyuan City, Taiwan; 4 Behavioral Sciences Research Center, Life Style Institute, Baqiyatallah University of Medical Sciences, Tehran, Iran; 5 Spiritual Health Research Center, Research Institute for Health Development, Kurdistan University of Medical Sciences, Sanandaj, Iran

**Keywords:** Psychometric evaluation, Diabetes, Diabetes foot, Self-care, Diabetes foot self-care

## Abstract

**Background:**

Diabetes foot self-care is one of the self-management behaviors of diabetic patients leading to a reduction in the incidence of pressure ulcers and amputation. Having a valid, reliable, simple and comprehensive tool is essential in measuring the self-care behavior of diabetic patients. The aim of this study was to evaluate the psychometric properties of the Farsi version of the diabetes foot self-care bahavior scale (DFSBS) in Iran.

**Methods:**

In this cross-sectional and methodological study, 500 patients with type 2 diabetes were recruited by convenience sampling. Construct validity was assessed by exploratory factor analysis (over 300 patients) and confirmatory factor analysis (over 200 patients). Internal consistency was calculated by Cronbach’s alpha coefficient and its stability was calculated by intraclass correlation coefficient (ICC).

**Results:**

In the exploratory factor analysis, two self-care factors related to feet and shoes were extracted which had specific values of 38.49 and 1.24, respectively, and were able to account for 56.22% of the total self-care variance of diabetes foot. Confirmatory factor analysis had excellent fit model. The internal consistency and ICC of the whole instrument were 0.83 and 0.791 (95% CI: 0.575–0.925; *P* < 0.001), respectively.

**Conclusions:**

The Farsi version of DFSBS (F-DFSBS) has good validity and reliability, and due to its appropriate psychometric properties, this tool can be used in future studies.

## Background

Diabetes is the most common chronic metabolic disease that has now become a silent epidemic [1]. The prevalence of diabetes and its subsequent adverse effects has been on the rise around the world. According to the World Health Organization (WHO), in 2017, nearly 425 million people had diabetes and the number of these patients is expected to increase significantly due to population growth, obesity, aging and urbanization [2]. The number of people with diabetes in 2019 was 463 million in the world, which is expected to increase to 700 million by 2045 [3]. Complications of diabetes lead to decreased quality of life, increased financial costs, morbidity and mortality [4]. Patients with diabetes are at risk for macrovascular (cardiovascular and cerebrovascular disease) and microvascular (retinopathy, nephropathy, and neuropathy) complications [5, 6]. Neuropathy often leads to foot ulcers and amputations, accounting for 50 to 75% of non-traumatic amputations [7, 8], and one foot is amputated every 30 s due to diabetic peripheral neuropathy [9]. According to the meta-analysis of Sobhani et al., the prevalence of diabetic peripheral neuropathy in Iran is 53% [10]. It is estimated that 15% of patients with diabetes experience diabetes foot ulcers during their lifetime [11]. The disability of diabetic peripheral neuropathies results in the limitation of daily activities and performance in patients’ family and social roles [12]. Persistent diabetic foot ulcers reduce not only the quality of life of patients but also the quality of life of their companions [13].

Diabetes is a chronic disease that requires lifelong self-care behaviors, and because more than 95% of diabetic care is provided by patients themselves, successful control of the disease relies upon the self-care of these patients [14]. Patients with diabetes need lifelong self-care to prevent short-term and long-term complications of diabetes and improve quality of life [15]. Proper foot care prevents diabetes foot ulcers and subsequent amputations [16]. Proper prevention, patient education and self-care could reduce the risk of diabetes foot complications by 50 to 85% [17]. Despite the importance of diabetic foot self-care, the results of various studies have indicated that 20% of patients with diabetes never examine their feet during the week, and 15% never dry their toes after washing their feet [18–20]. Although all patients at risk for diabetic foot ulcers (patients with diabetic peripheral neuropathy) should examine their feet thoroughly on a daily basis, only half of them do so [21, 22].

There are many tools to examine and measure self-care in patients with diabetes [23–38], but few of them exclusively examine the self-care behaviors associated with and in most of these tools, self-care is examined in general. Instruments that measure diabetes foot self-care either have a large number of items that reduce the tendency to respond [20] or have not undergone a psychometric process [39]. One of the valid and reliable tools with low number of items (7 items) is Diabetes Foot Self-care Behavior Scale (DFSBS) tool designed by Chin and Huang in Taiwan. The first 4 questions deals with the examination of the soles of the feet, toes, washing and drying them during the week, and the answers are from zero to 7 which are” No day (score1), 1 to 2 days (score2), 3 to 4 days (score3), 5 to 6 days (score4) and the whole week (score5) respectively. The score of this section varies between 4 and 20. The other three questions are about using lotions and examining shoes, the answers to which are arranged in the form of a five-point Likert scale from Never (score 1) to Always (score 5). The score of this section is between 3 to 15 and the total score range of the questionnaire is 7 to 35, the higher the score, the higher the self-care. Based on the above, this study was conducted to investigate the psychometric properties of the Farsi version of diabetes foot self-care scale.

## Methodology

### Design of the study

This cross-sectional study was performed with the aim of psychometric evaluation of the Farsi version of the DFSBS instrument on patients with diabetes referred to Shahrekord Diabetes Center in 2020.

### Sample

Estimation of sample size in psychometric studies is somewhat controversial. Some believe that exploratory factor analysis requires 5 to 10 samples per item, and some state that the sample size of 150 to 300 is appropriate, 300 to 500 is good, and over 500 is excellent. Also, for confirmatory factor analysis, the sample size should not be less than 200 people [40–42]. Accordingly, for exploratory and confirmatory factor analysis, 300 and 200 patients with diabetes were selected, respectively.

### Inclusion and exclusion criteria

Patients who had been diagnosed with the disease for more than a year and had a record in the diabetes unit were included in the study. Patients with untreated diabetic foot ulcers, patients with cognitive disorder, and patients who were unable to communicate were not included in the study.

### Translation process

We used forward-backward translation procedure to translate the English version of the questionnaire into Farsi [43]. At first, the questionnaire was translated from Farsi to English by two translators independently and the final Persian version was compiled by comparing and combining the two translated versions. In the next step, the Farsi version was given to two bilingual translators to translate it into English, and the final version was prepared by reviewing the two translated versions. In both Forward and Backward stages, one of the translators was familiar with medical terms, but the other was completely unfamiliar. Any disagreements at this stage were resolved through team discussions and consultation with the original tool designers.

### Face validity and content validity

Face validity and content validity were performed qualitatively. To assess face validity, a questionnaire was sent to ten literate patients with diabetes and they were asked to read it aloud and inform us of any ambiguity or difficulty in understanding the items. For content validity, five experts (2 nurses, 1 orthopedic specialist and 2 infectious disease specialists) were asked to review the Farsi version of the questionnaire in terms of content. The ceiling and floor effect was also investigated. There is a floor or ceiling effect if more than 15% of respondents have the lowest or highest possible score, respectively [44]. If floor or ceiling effects are present, it is likely that extreme items are missing in the lower or upper end of the scale, indicating insufficient content validity [45].

### Data analysis

Exploratory factor analysis was conducted to evaluate the construct validity using the data of first 300 participants in this study. Adequacy of sampling was assessed with Kaiser-Meyer-Olkin (KMO). Sampling adequacy index of 0.7 to 0.8 is considered good and 0.8 to 0.9 regarded as excellent. Bartlett test of sphericity was used to evaluate the significance of the correlation matrix between variables. Extraction of latent factors was performed by PASW v18 (SPSS Inc. Chicago, IL, USA) using maximum likelihood and Promax rotation. The cut-off factor was considered to be 0.30.

Then the data of remaining 200 participants were used to perform confirmatory factor analysis. At this stage, root mean square error of approximation (RMSEA), comparative fit index (CFI), goodness of fit (GFI), incremental fit index (IFI), normed fit index (NFI), relative fit-index (RFI), adjusted goodness of fit index (AGFI), parsimonious normed fit index (PNFI) were evaluated with LISREL software. Internal consistency with Cronbach’s alpha coefficient and instrument stability were calculated by intraclass correlation coefficient (ICC) with two-way mixed effects model and absolute agreement with 95% confidence interval, which is acceptable above 0.75 [15].

### Ethical considerations

This study was conducted in accordance with the Declaration of Helsinki and was approved by the Ethics Committees of the Shahrekord Universities of Medical Sciences (Iran) with number (IR.SKUMS.REC.1399.133). The participants were previously informed about the characteristics of the study. They were all asked to complete a questionnaire and to provide signed consent to confirm the participation in the study.

## Results

The sample under investigation consisted of 500 diabetes patients with a mean age of 53 ± 17.8 years with a mean duration of disease of 7.5 ± 8.2 years. The majority of the studied samples were female (62%), married (83.6), had university education (40.6%), housewife and unemployed (43.2%). More information is provided in Table 1.

**Table 1:** Mean score of diabetes foot self-care by demographic variables

Variable	Number	Percent	Mean Score
Gender			
Male	190	38	18.65 ± 4.72
Female	310	62	19.22 ± 4.81
Educational Level			
Illiterate	92	18.4	17.25 ± 3.77
Elementary/Junior high school	105	21	17.66 ± 3.93
High school/ Diploma	100	20	19.44 ± 5.40
University degree	203	40.6	20.30 ± 4.89
Employment Status			
Housewife/ unemployed	216	43.2	18.37 ± 4.27
Retired	44	8.8	19.07 ± 5.65
Employed	150	30	19.86 ± 4.82
Self-employed	39	7.8	18.08 ± 5.68
Others	51	10.2	19.80 ± 4.93
Marital Status			
Married	418	83.6	18.84 ± 4.66
Single	82	16.4	19.85 ± 5.30
Foot ulcer history			
Yes	54	10.8	18.90 ± 4.74
No	446	89.2	19.90 ± 5.02
Medicine			
Tablet	344	68.8	18.81 ± 4.81
Insulin	118	23.6	18.77 ± 4.05
Tablet and Insulin	38	7.6	21.52 ± 5.91

The ceiling and floor effect was %5 and 4% for the first dimension and 3 and 1% for the second dimension, respectively, which is acceptable.

### Construct validity

#### Exploratory factor analysis

Face validity and qualitative content validity were confirmed and applied after reviewing and applying the opinions of patients and qualified specialists (2 nurses, 1 orthopedic specialist and 2 infectious disease specialists). KMO was 0.806 and Bartlett sphericity test was significant (Chi-Square = 1217.72, df = 21, *p* = 0.0001). Exploratory factor analysis was performed by Maximum likelihood method and Promax rotation. The analysis resulted in the extraction of two factors (self-care in relation to feet and shoes) which together explained 56.2% of the total variance. The first factor with 4 items (items 2, 4, 3 and 1) explains 44.786% of the variance of diabetes foot self-care and the second factor with three items (items 5, 6 and 7) explains 43.47% of the variance of diabetes foot self-care. The Eigen values of the first and second factors were 3.485 and 1.241, respectively. The results of the exploratory factor analysis are presented in detail in Table 2.

**Table 2:** Exploratory factor analysis of Persian version of the DFSBS

Factors	Items	Factor loading	h^2^	% variance	Eigen value	Cronbach alpha
1	2- I (my caregiver) examine between my toes	0.881	0.728	44.786	3.485	0.88
	4- I (my caregiver) dry between my toes after washing	0.826	0.729			
	3- I (my caregiver) wash between my toes	0.789	0.637			
	1- I (my caregiver) examine the soles of my feet	0.763	0.588			
2	5- If I feel dry on the skin of my feet, I (my caregiver) apply moisturizing cream on it.	0.897	0.760	11.437	1.241	0.605
	6- Before I wear my shoes, do I check the inside of my shoes (or does my caregiver do this)?	0.562	0.401			
	7-It takes me a while to feel comfortable in the new shoes I buy.	0.315	0.094			

**Fig. 1:**
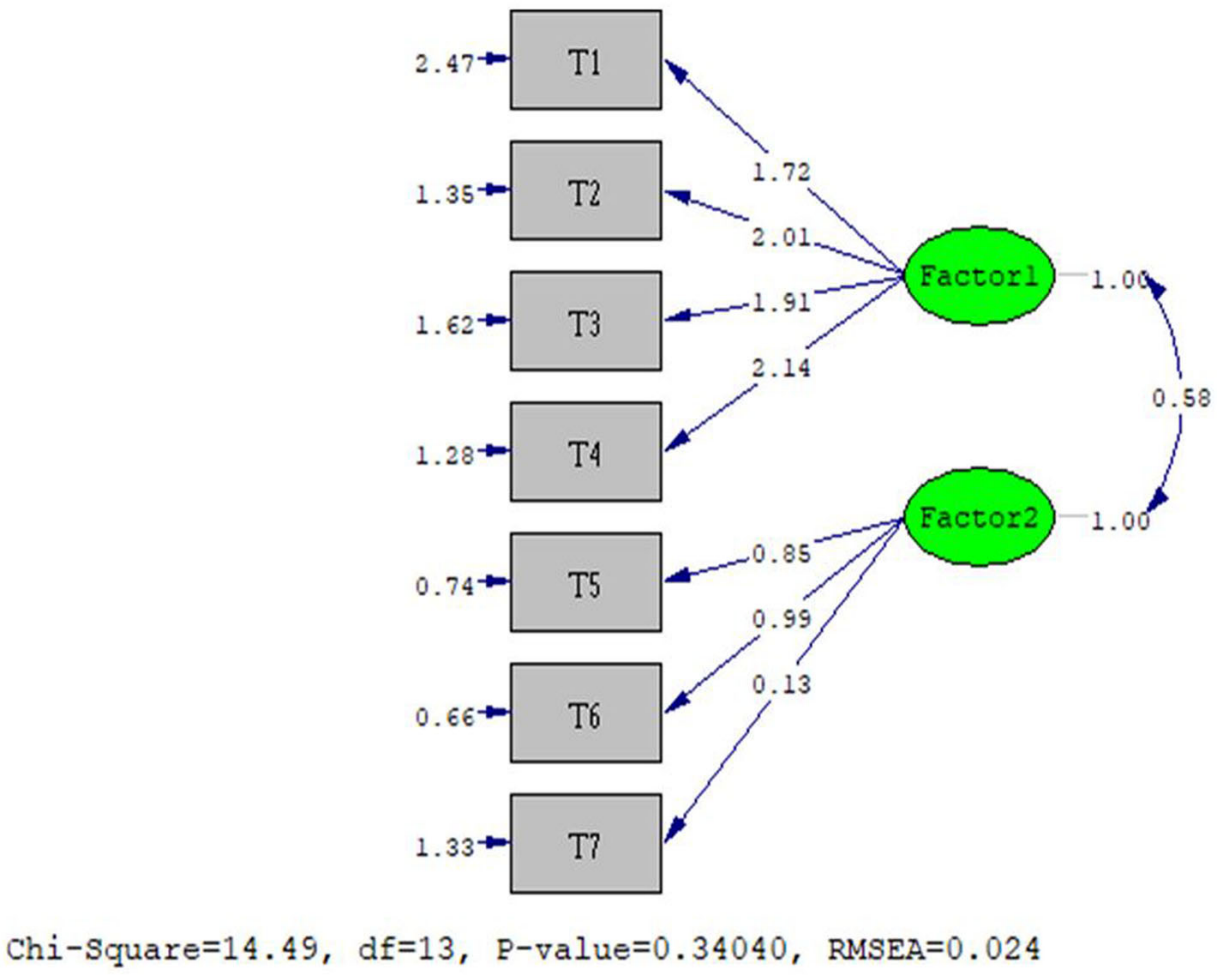
Final Model

In confirmatory factor analysis, the results of goodness of fit test of chi-square were obtained (*p* = 0.01, X2 = 44.31). As shown in Fig. 1, fit indicators were good: Fit indicators were good: Root Mean Square Error of Approximation (RMSEA) = 0.024; Comparative Fit Index (CFI): 0.98; Goodness of Fit Index (GFI): 0.98; Incremental Fit Index (IFI): 0.97; Adjusted Goodness of Fit Index (AGFI): 0.96; Parsimonious Normed Fit Index (PNFI): 0.78; and Minimum Discrepancy Function by Degrees of Freedom divided (CMIN/DF) = 8.4.

Regarding the internal consistency of the questionnaire, the first and second factors based on Cronbach’s alpha coefficient were 0.828, 0.88 and 0.605, respectively. The ICC of the whole instrument was 0.791 (95% CI: 0.575–0.925; *P* < 0.001).

## Discussion

The questionnaire included seven items examining the sole of the foot and between the toes, washing between the toes, drying between the toes after washing, using lotion, examining inside the shoe as well as feeling comfortable in the shoe. In the original version, the KMO was 0.72 and Bartlett test of sphericity was 475.86 (*p* < 0.001) [20] and in the Persian version, KMO was 0.806 and Bartlett test was 1217.725. Although the Scree plot diagram had two elbows in the exploratory factor analysis of the original version, the designers considered the questionnaire to be a single factor that explained 39% of the total variance. In the Persian version, two factors were extracted that explained 56/233% of the total variance, which is more than the original version. The highest factor load (0.8) was related to item 2 (I (my caregiver) examine between the toes of my feet) and the lowest factor load was related to item 7 (I break in new shoes slowly) which was in line with the results of our exploratory factor analysis. In the original version, in known-groups validity, the mean score of diabetes foot self-care in patients with a previous history of diabetes foot ulcer was significantly higher than patients without a history of diabetes foot ulcer. In the present study, the mean self-care score of diabetes foot was higher in patients with academic education. It seems that as education increases, patients’ awareness of self-care behaviors increases.

The first factor was self-care in relation to the feet and the second factor was self-care in relation to shoes. The first factor explained more of the overall variance of this questionnaire than the second factor. The World Health Organization state in a statement that the reduction of 50% of the related diabetes gangrene amputations has been required owing to the belief that prevention of the diabetic foot is possible provided that good patient management is present [46]. In a study in Tanzania, 87% of diabetes patients reported that they never examined their feet, and 66% declared they were not interested in learning more about foot care [47]. The second factor, which referred to shoes and lotions, explained a lower variance of diabetes foot self-care than the first factor. Self-care of the feet (examination of the soles of the feet, toes, washing and drying the feet) seems to be more significant than shoes and lotions.

In a study by Bell et al., 54% of patients reported not examining inside their shoes before wearing them [18]. Wearing inappropriate shoes or walking barefoot could cause local mechanical repetitive stresses on the feet, which might cause ulcers, so diabetics should wear appropriate shoes to put less pressure on their feet [48]. It is even recommended that patients wear shoes both outdoors and indoors, although most do not do so at home [49]. In general, it seems that the prevention of diabetes foot ulcers will be possible only if patients engage in self-care behaviors.

The internal consistency of the original version based on Cronbach’s alpha coefficient was 0.73 and the consistency of the questionnaire with a two-week interval was 0.92. In the present study, the internal consistency of the whole questionnaire based on Cronbach’s alpha coefficient was 0.828, which is acceptable [49]. The results of exploratory and confirmatory factor analysis on the Persian version of the Diabetes Foot Self-Care Questionnaire with 7 items indicated that the structure of this questionnaire has good validity and reliability.

## Conclusion

The Farsi version of the diabetes foot self-care scale is valid and reliable, and the small number of items permits patients to easily understand and respond to it. This scale can be used to assess the self-care status of patients’ foot self-care and to plan educational and care interventions to promote self-care.

## Data Availability

The data that support the findings of this study are not publicly available. Data are however available from the authors upon reasonable request and with permission of Shahrekord University of Medical Sciences.

## Abbreviations

DFSBS: Diabetic foot self-care behavior scale
ICC: Intraclass correlation coefficient (ICC)
KMO: Kaiser-Meyer-Olkin
WHO: World Health Organization
